# Association between afterhours admission to the intensive care unit, strained capacity, and mortality: a retrospective cohort study

**DOI:** 10.1186/s13054-018-2027-8

**Published:** 2018-04-17

**Authors:** Adam M. Hall, Henry T. Stelfox, Xioaming Wang, Guanmin Chen, Danny J. Zuege, Peter Dodek, Allan Garland, Damon C. Scales, Luc Berthiaume, David A. Zygun, Sean M. Bagshaw

**Affiliations:** 1 Department of Critical Care Medicine, Faculty of Medicine and Dentistry, University of Alberta and Alberta Health Services, 2-124E Clinical Sciences Building, 8440-112 ST NW, Edmonton, T6G 2B7 Canada; 2 Department of Critical Care Medicine, Cumming School of Medicine, University of Calgary and Alberta Health Services, Calgary, Canada; 3 Critical Care Strategic Clinical Network, Alberta Health Services, Edmonton, Canada; 4 Research Facilitation, Research Analytics (DIMR), Alberta Health Services, Edmonton, Canada; 5 Division of Critical Care Medicine and Center for Health Evaluation and Outcome Sciences, St. Paul’s Hospital and University of British Columbia, Vancouver, Canada; 6 Section of Critical Care Medicine, Department of Medicine, University of Manitoba, Winnipeg, Canada; 7 Department of Critical Care Medicine, University of Toronto and Sunnybrook Health Sciences Centre, Toronto, Canada

**Keywords:** Intensive care unit, Afterhours admission, ICU mortality, APACHE II score

## Abstract

**Background:**

Admission to the intensive care unit (ICU) outside daytime hours has been shown to be variably associated with increased morbidity and mortality. We aimed to describe the characteristics and outcomes of patients admitted to the ICU afterhours (22:00–06:59 h) in a large Canadian health region. We further hypothesized that the association between afterhours admission and mortality would be modified by indicators of strained ICU capacity.

**Methods:**

This is a population-based cohort study of 12,265 adults admitted to nine ICUs in Alberta from June 2012 to December 2014. We used a path-analysis modeling strategy and mixed-effects multivariate regression analysis to evaluate direct and integrated associations (mediated through Acute Physiology and Chronic Health Evaluation (APACHE) II score) between afterhours admission (22:00–06:59 h) and ICU mortality. Further analysis examined the effects of strained ICU capacity and varied definitions of afterhours and weekend admissions. ICU occupancy ≥ 90% or clustering of admissions (≥ 0.15, defined as number of admissions 2 h before or after the index admission, divided by the number of ICU beds) were used as indicators of strained capacity.

**Results:**

Of 12,265 admissions, 34.7% (*n* = 4251) occurred afterhours. The proportion of afterhours admissions varied amongst ICUs (range 26.7–37.8%). Patients admitted afterhours were younger (median (IQR) 58 (44–70) vs 60 (47–70) years, *p* < 0.0001), more likely to have a medical diagnosis (75.9% vs 72.1%, *p* < 0.0001), and had higher APACHE II scores (20.9 (8.6) vs 19.9 (8.3), *p* < 0.0001). Crude ICU mortality was greater for those admitted afterhours (15.9% vs 14.1%, *p* = 0.007), but following multivariate adjustment there was no direct or integrated effect on ICU mortality (odds ratio (OR) 1.024; 95% confidence interval (CI) 0.923–1.135, *p* = 0.658). Furthermore, direct and integrated analysis showed no association of afterhours admission and hospital mortality (*p* = 0.90) or hospital length of stay (LOS) (*p* = 0.27), although ICU LOS was shorter (*p* = 0.049). Early-morning admission (00:00–06:59 h) with ICU occupancy ≥ 90% was associated with short-term (≤ 7 days) and all-cause ICU mortality.

**Conclusions:**

One-third of critically ill patients are admitted to the ICU afterhours. Afterhours ICU admission was not associated with greater mortality risk in most circumstances but was sensitive to strained ICU capacity.

**Electronic supplementary material:**

The online version of this article (10.1186/s13054-018-2027-8) contains supplementary material, which is available to authorized users.

## Background

Patients may suffer clinical deterioration and develop critical illness at unpredictable times, commonly outside conventional daytime hours. The importance of early resuscitation in critical illness is well described. Adequate early resuscitation can improve outcomes in sepsis [1], vascular emergencies [2–5], trauma [6], and cardiac arrest [7, 8]. As such, improving outcomes for critically ill patients necessitates that critical care services be accessible 24 h a day and 7 days a week [9–11].

While numerous organizational factors likely influence access to critical care services and outcomes for critically ill patients, prior studies have suggested that intensive care unit (ICU) admission occurring outside conventional work hours [12–16] or on weekends [17, 18] is associated with greater risk for major morbidity and mortality. Prior data on this issue have been discordant, with some studies reporting increased risk of mortality for ICU admission occurring afterhours [19], while others, including two recent systematic reviews [20, 21], suggested no incremental hazard.

The association between patient outcomes and afterhours ICU admission may be partly influenced by ICU organizational factors (i.e., staffing models, capacity) that are not patient related, and as such may be highly variable across health systems. Knowledge of avoidable ICU organization issues that may impact patient care and outcome, such as the effect of time of ICU admission, are important to understand to enable health system improvement, to guide procedure and policy development, and for workplace and capacity planning.

We hypothesized, in part due to data suggesting that critical care services in our large health region commonly experience strained capacity [22], that patients admitted to the ICU afterhours would suffer greater risk for death compared with daytime hours, and that this association would be modified by indicators of strained capacity. Accordingly, we performed a population-based study to evaluate the association between afterhours ICU admission and mortality among adult critically ill patients.

## Methods

This study was approved by the Human Research Ethics Board at the University of Alberta prior to commencement (File # Pro00046184). The need for written informed consent was waived.

### Study design, setting, and population

This was a population-based cohort study utilizing routinely captured data evaluating all adult (age ≥ 15 years) patients (*n* = 12,265) admitted to nine ICUs (median (IQR) number of ICU beds 24 (18–28)) in Alberta, Canada, from June 2012 to December 2014. All ICUs were mixed medical/surgical units in two major cities: Calgary (four units) and Edmonton (five units). Of these, two were classified as academic/quaternary, two as academic/tertiary, and five as metropolitan/community ICUs. All included ICUs utilized a “closed” model and were staffed by certified intensivists, who were present during the daytime hours each day and available afterhours on call, with supplemental in-house coverage by clinical associates or resident trainees.

### Data sources

Data were analyzed from eCritical Alberta, a provincial clinical information system, data warehouse, and clinical analytics system [22]. eCritical is a bedside interdisciplinary electronic documentation system (MetaVision™; iMDsoft) which captures demographic, diagnostic/case-mix (i.e., comorbidity, diagnostic classification, surgical status, Acute Physiology and Chronic Health Evaluation (APACHE) II and III score), laboratory, and device (physiologic monitors, ventilators, renal replacement therapy, use of vasoactive medications) data. TRACER is a comprehensive, multimodal, and integrated data repository and clinical analytics system. The eCritical Alberta program includes rigorous methods of data quality assurance, including auditing of high value data. eCritical systems have been used previously to facilitate health services research [22–24]. Missing data in our cohort were uncommon (< 3% for any data variable included in the analyses). For our primary exposure (afterhours admission) and primary outcome (ICU mortality), there were no missing data elements.

### Main exposures and outcomes

The primary exposure was ICU admission afterhours, defined as occurring between 22:00 and 06:59 h. The primary outcome was ICU mortality. Secondary outcomes included ICU mortality within 30 h, 3 days, and 7 days following admission, in-hospital mortality, and ICU and hospital length of stay (LOS). Other study variables included age, sex, case mix (e.g., diagnostic classification, surgical status, comorbidities, and Charlson comorbidity index), admission source (i.e., emergency department (ED), operating theater (OR), hospital ward), and ICU site (i.e., location, hospital type). The associations between afterhours admission and mortality were further evaluated with indicators of strained ICU capacity, including instantaneous bed occupancy at ICU admission [22], and clustering of admissions (quantified by number of admissions 2 h before or after the index admission, divided by the number of funded ICU beds).

### Statistical methods

#### Descriptive analysis

Data were initially explored descriptively. Normally or near normally distributed data, confirmed by histogram, are reported as means with standard deviations (SDs) and compared by Student’s *t* test. Nonnormally distributed continuous data are reported as medians with interquartile ranges (IQRs) and were compared by Wilcoxon–Mann–Whitney *U* test. Categorical variables were compared using the chi-squared test.

#### Path-analysis modeling

Additional file 1 outlines our mediation analysis model of the direct and indirect (mediated through APACHE II score) relationship between afterhours admission and ICU mortality. First, we estimated the association between afterhours admission and APACHE II score by a random-effects multivariate linear regression model, adjusted for demographics (e.g., age, sex), Charlson comorbidity index, case mix (e.g., diagnostic classification, surgical status), instantaneous bed occupancy at ICU admission, and ICU site (e.g., location, hospital type) (Additional file 2). We assumed that intercepts for each of the nine ICU sites were random, implying that different ICUs have different illness severity levels. This model allows calculation of the association between afterhours admission and APACHE II scores. We then estimated the effects of afterhours admission and APACHE II score on ICU mortality by a random-effects multivariate logistic regression model (Additional files 3, 4, 5, 6, 7, and 8). ICU type was again used as random-effects predictor, and the model was similarly adjusted as in the linear model. Customized variable selection was adopted to produce sparse models for the multivariate modeling analyses [25]. All of the modeling analyses were conducted in SAS (release 9.4; SAS Institute, Cary, NC, USA). Lastly, simulation experiments (1 million replicates) were conducted in R Core [26] to estimate the total combined effect by integrating both the direct and indirect effects of afterhours admission on ICU mortality. Random-effects multivariate Poisson regression was used to evaluate the association between afterhours admission, APACHE II score, and hospital LOS (Additional file 9).

#### Sensitivity analysis

Sensitivity analysis was performed using the same path-analysis model with modified definitions of afterhours admission including the following: early morning admission, defined as 00:00–06:59 h; early morning admission on a weekend or statutory holiday (non-work day); early morning admission in winter (October–March); early morning admission with high bed occupancy (≥ 90%, ≥ 95%), and afterhours admission with clustering of admissions.

## Results

### Afterhours ICU admission

**Fig. 1:**
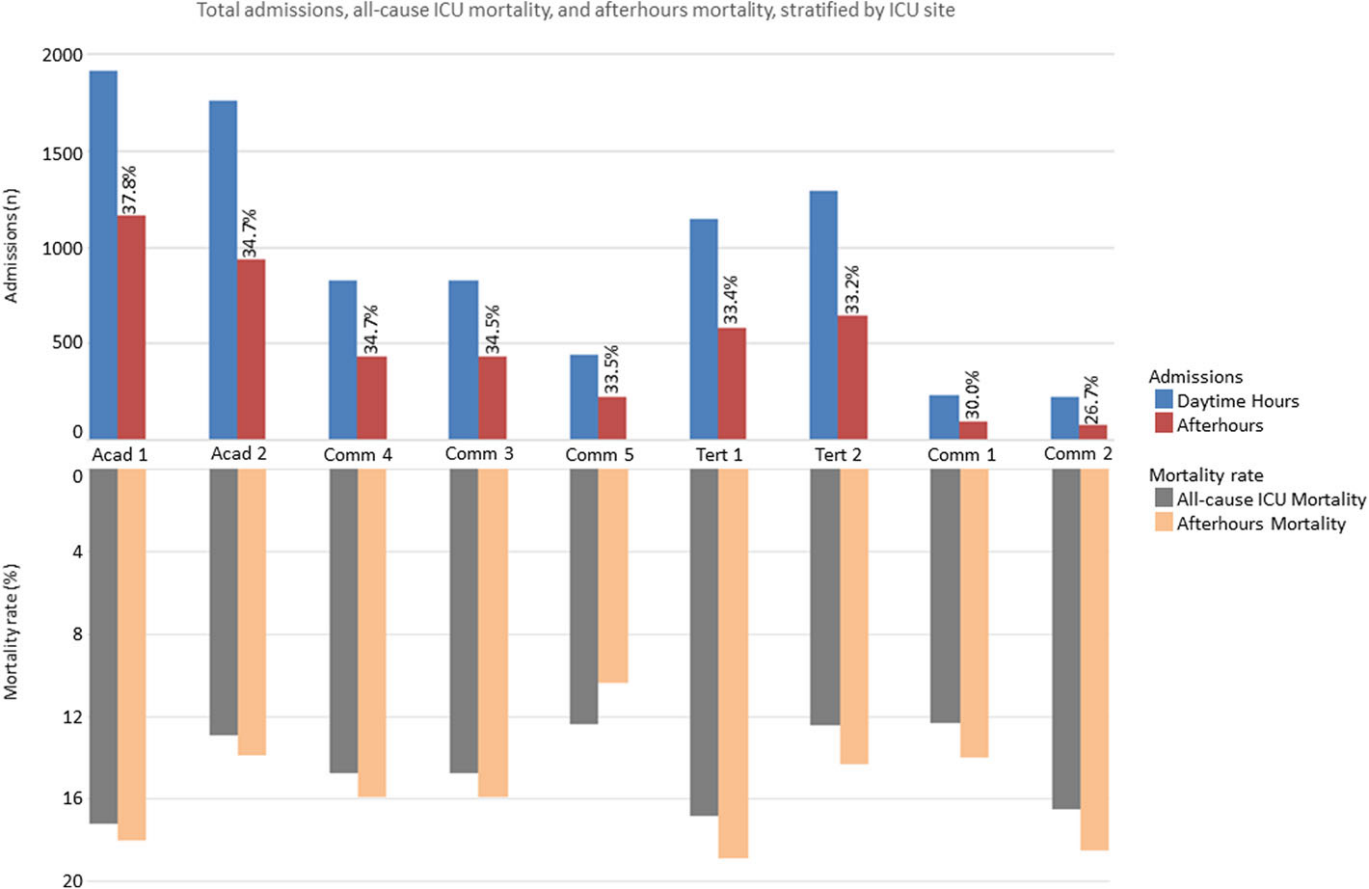
Total admissions, all-cause ICU mortality, and afterhours mortality, stratified by ICU site. Site-specific total admissions (*n*) and mortality (%), stratified by daytime hours and afterhours admission time. Sites ordered (left to right) by decreasing proportion of afterhours admissions to total admissions, with percentage of total admissions occurring afterhours indicated numerically. Acad academic hospital, Comm community hospital, ICU intensive care unit, Tert tertiary hospital

Of 12,265 admissions to the nine ICUs, 34.7% (*n* = 4251) occurred afterhours. Afterhours admission was most common in academic units (Table 1). There was significant variability across units (range 26.7–37.8%; Figure 1). Most afterhours admissions were referred from the ED (46.4%), hospital ward (27.1%), and operating theater (16.9%), respectively. Afterhours admissions were also associated with greater occupancy, but less clustering of ICU admissions. Of afterhours admissions, 30.6% (*n* = 1302) were admitted on weekends/holidays, 47.0% (*n* = 1998) were during winter months (October–March), while 70.9% (*n* = 3016) were between 00:00 and 06:59 h, respectively. Patients admitted afterhours were younger, mostly nonoperative, and had greater APACHE II scores (Table 2).

**Table 1:** Summary of ICU characteristics, stratified by time of ICU admission

Characteristic	Total (*n* = 12,265, 100%)	Workhours (*n* = 8014, 65.3%)	Afterhours (*n* = 4251, 34.7%)	*p* value
Location, *n* (%)				0.012
Calgary	6732 (54.9)	4333 (54.1)	2399 (56.4)	
Edmonton	5533 (45.1)	3681 (45.9)	1852 (43.6)	
Hospital type				0.0009
Academic	5776 (47.1)	3676 (45.9)	2100 (49.4)	
Tertiary	3664 (29.9)	1895 (23.7)	930 (21.9)	
Community	2825 (23.0)	2443 (30.5)	1221 (28.7)	
ICU, *n* (%)				< 0.0001
Academic 1	3083 (25.1)	1918 (23.9)	1165 (27.4)	
Community 1	333 (2.7)	233 (2.9)	100 (2.4)	
Community 2	303 (2.5)	222 (2.8)	81 (1.9)	
Tertiary 1	1728 (14.1)	1150 (14.3)	578 (13.6)	
Tertiary 2	1936 (15.8)	1293 (16.1)	643 (15.1)	
Community 3	1258 (10.3)	824 (10.3)	434 (10.2)	
Community 4	268 (2.2)	175 (2.2)	93 (2.2)	
Community 5	663 (5.4)	441 (5.5)	222 (5.2)	
Academic 2	2693 (22.0)	1758 (21.9)	935 (22.0)	
Number of ICU beds, median (IQR)	24 (18–28)	24 (10–28)	25 (18–28)	0.0001
Admitted/transferred from				< 0.0001
Emergency department	4823 (39.3)	2851 (35.6)	1972 (46.4)	
PACU/operating theater	2398 (19.6)	1680 (21.0)	718 (16.9)	
Post procedure^a^	44 (0.4)	38 (0.5)	6 (0.1)	
Other critical care unit	313 (2.6)	238 (3.0)	75 (0.2)	
Ward transfer	3681 (30.0)	2528 (31.5)	1153 (27.1)	
Outside hospital	428 (3.5)	268 (3.3)	160 (3.8)	
Other^b^	358 (2.9)	263 (3.3)	95 (2.2)	
Unspecified^c^	221 (1.8)	149 (1.9)	72 (1.7)	
Admitted on weekend/holiday	3471 (28.3)	2169 (27.1)	1302 (30.6)	< 0.0001
Admitted during winter (October–March)	5799 (47.3)	3801 (47.4)	1998 (47.0)	0.66
Bed occupancy rate, median (IQR)	85.7 (79.9–92.3)	85.7 (76.0–92.3)	86.7 (77.8–92.3)	0.040
Occupancy < 90%, *n* (%)	7828 (63.8)	5141 (64.2)	2687 (63.2)	0.20
Occupancy ≥ 90%, *n* (%)	4177 (34.1)	2694 (33.6)	1483 (34.9)	0.20
Clustering of admissions, per bed^d^				
Clustering admission per bed < 0.15	10,252 (83.6)	6586 (82.2)	3666 (86.2)	< 0.0001
Clustering admission per bed ≥ 0.15	2013 (16.4)	1428 (17.8)	585 (13.8)	< 0.0001

*ICU* intensive care unit, *IQR* interquartile range, *PACU* postanesthetic care unit

^a^Unplanned admissions following bronchoscopy, endoscopy, cardiac catheterization, interventional radiology, etc.

^b^Patients’ admission source classified by admitting physician as “other”

^c^Unspecified data points were unavailable in our database

^d^Number of admissions in the 2 h before or after the index admission, divided by the number of funded ICU beds

**Table 2:** Baseline patient characteristics, stratified by time of ICU admission

Characteristic	Total (*n* = 12,265, 100%)	Workhours (*n* = 8014, 65.3%)	Afterhours (*n* = 4251, 34.7%)	*p* value
Age, median (IQR)	59 (46–70)	60 (47–70)	58 (44–70)	<.0001
Age category, *n* (%)				0.0022
< 65 years	7680 (62.6)	4934 (61.6)	2746 (64.6)	
65–74 years	2458 (20.1)	1674 (20.9)	784 (18.5)	
75–84 years	1696 (13.8)	1132 (14.1)	564 (13.3)	
≥ 85 years	426 (3.5)	270 (3.4)	156 (3.7)	
Sex, *n* (%)				0.29
Female	5112 (41.7)	3367 (42.0)	1745 (41.1)	
Male	7150 (58.3)	4644 (58.0)	2506 (59.0)	
System, *n* (%)				<.0001
Cardiovascular	2103 (17.3)	696 (16.5)	696 (16.5)	
Gastrointestinal	2044 (16.8)	677 (16.0)	677 (16.0)	
Genitourinary	449 (3.7)	136 (3.2)	136 (3.2)	
Hematology	65 (0.5)	24 (0.6)	24 (0.6)	
Metabolic/endocrine	246 (2.0)	85 (2.0)	85 (2.0)	
Musculoskeletal/skin	485 (4.0)	144 (3.4)	144 (3.4)	
Neurologic	1809 (14.9)	679 (16.1)	679 (16.1)	
Respiratory	3643 (29.9)	1216 (28.8)	1216 (28.8)	
Transplant	129 (1.1)	52 (1.2)	52 (1.2)	
Trauma	1211 (9.9)	519 (12.3)	519 (12.3)	
Surgery, *n* (%)				<.0001
Elective	1209 (9.9)	962 (12.0)	247 (5.8)	
Emergent	2054 (16.8)	1276 (15.9)	778 (18.3)	
Nonoperative	9002 (73.4)	5776 (72.1)	3226 (75.9)	
Class, *n* (%)				<.0001
Medical	7241 (59.0)	4701 (58.7)	2540 (59.8)	
Neurological	851 (6.9)	501 (6.3)	350 (8.2)	
Surgical	3101 (25.3)	2203 (27.5)	898 (21.1)	
Trauma without head injury	647 (5.3)	379 (4.7)	268 (6.3)	
Trauma with head injury	425 (3.5)	230 (2.9)	195 (4.6)	
Comorbidity disease, *n* (%)				
Chronic dialysis	423 (3.5)	273 (3.4)	150 (3.5)	0.72
Hepatic	1264 (10.3)	817 (10.2)	447 (10.5)	0.58
Neurologic	5298 (43.2)	3303 (41.2)	1995 (46.9)	<.0001
AIDS	65 (0.5)	42 (0.5)	23 (0.5)	0.90
Chronic heart failure	762 (6.2)	512 (6.4)	250 (5.9)	0.27
Respiratory	1415 (11.5)	942 (11.8)	473 (11.1)	0.30
Metastatic/leukemia/lymphoma	791 (6.5)	543 (6.8)	248 (5.8)	0.043
Immune suppression	1095 (8.9)	736 (9.2)	359 (8.5)	0.17
Diabetes	2280 (18.6)	1535 (19.2)	745 (17.5)	0.027
Cirrhosis	785 (6.4)	526 (6.6)	259 (6.1)	0.31
Cardiovascular	5752(46.9)	3739 (46.7)	2013 (47.4)	0.47
Digestive	2210 (18.0)	1449 (18.1)	761 (17.9)	0.80
Acute renal	2869 (23.4)	1900 (23.7)	969 (22.8)	0.26
Charlson Index, median, (IQR)	1 (0-2)	1 (0-2)	1 (0-2)	<.0001
Admission APACHE II score, mean (SD)	20.2 (8.4)	19.9 (8.3)	20.9 (8.6)	<.0001

*AIDS* acquired immune deficiency syndrome, *APACHE* acute physiology and chronic health evaluation, *ICU* intensive care unit, *IQR* interquartile range, *SD* standard deviation

### Association between afterhours admission and APACHE II score

In multivariate analysis, afterhours admission was associated with significantly higher APACHE II scores (estimate (SE) 0.78 (0.13), *p* < 0.0001) (Additional file 2). Female sex, emergent surgical admission, diagnostic category, and higher occupancy were also associated with higher APACHE II scores, while elective surgical status was associated with lower APACHE II scores.

### Association between afterhours admission and ICU mortality

**Fig. 2:**
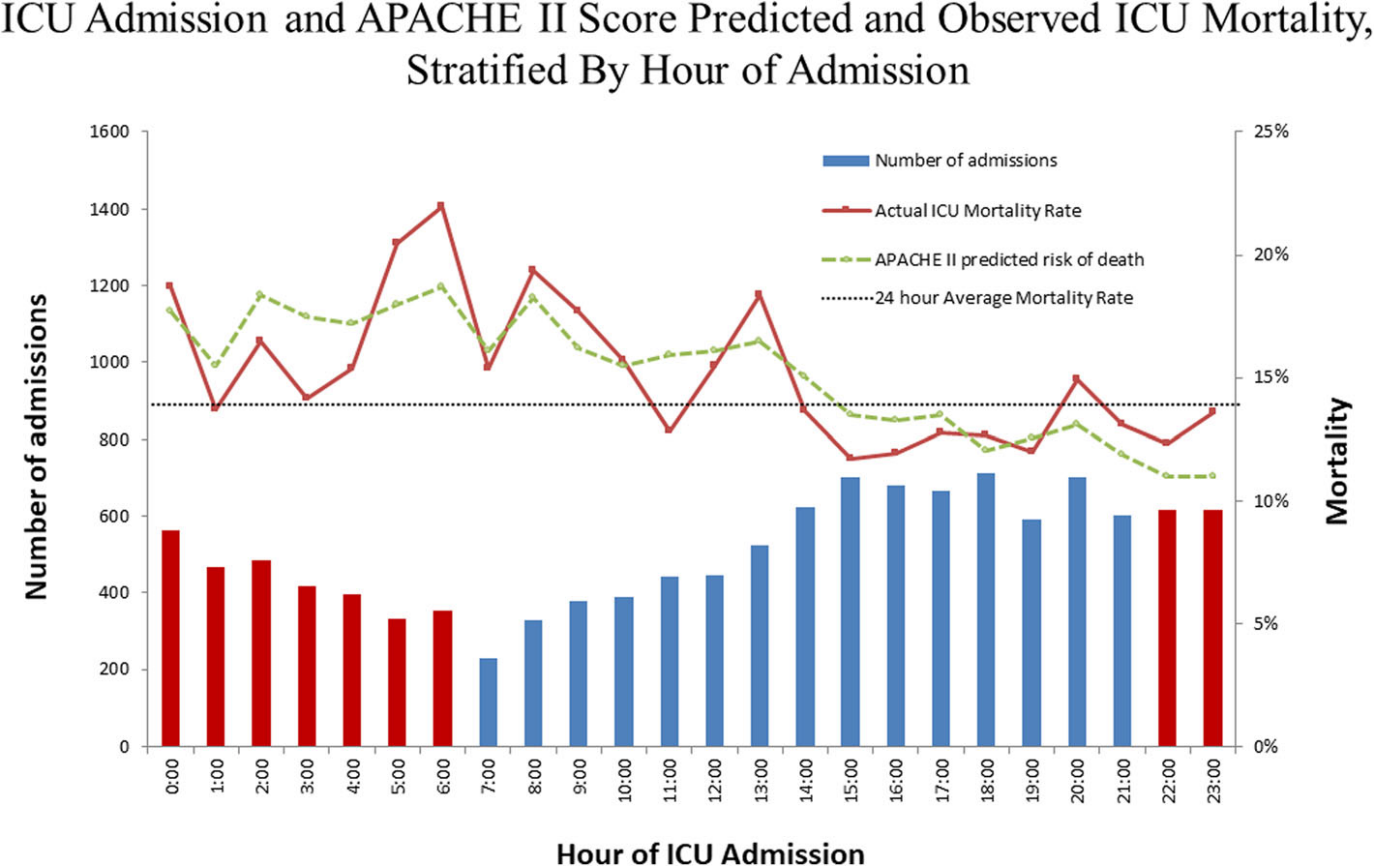
ICU admission and APACHE II score predicted and observed mortality rates, stratified by hour of ICU admission. Histogram demonstrating number of admissions by hour with red and blue bars representing afterhours and daytime hours, respectively. Line graph shows APACHE II predicted risk of death, with superimposed actual ICU mortality rate, by hour. APACHE Acute Physiology And Chronic Health Evaluation, ICU intensive care unit

ICU mortality was 14.7% (*n* = 1800). Unadjusted ICU mortality was greater for afterhours compared with daytime hours admission (15.9% vs 14.1%; OR 1.15; 95% CI 1.04–1.28, *p* = 0.007) (Table 3; Figure 2). This effect was persistent across variable durations following ICU admission. However, after multivariate adjustment, there was no significant direct or integrated effect (mediated through APACHE II score) of afterhours admission on ICU mortality (Table 4).

**Table 3:** Mortality and length of stay stratified by time of ICU admission

Outcome	Workhours (*n* = 8014)	Afterhours (*n* = 4251)	Absolute difference	Unadjusted OR (95% CI)	*p* value
Death within 30 h (*n*, %)	364 (4.5%)	238 (5.6%)	1.28%	1.25 (1.05–1.47)	0.0099
Death within 3 days (*n*, %)	585 (7.3%)	371 (8.7%)	1.43%	1.21 (1.06–1.39)	0.0050
Death within 7 days (*n*, %)	771 (9.6%)	495 (11.6%)	2.02%	1.24 (1.10–1.40)	0.0005
Death in ICU (*n*, %)	1127 (14.1%)	675 (15.9%)	1.82%	1.15 (1.04–1.28)	0.0069
Death in hospital (*n*, %)	1670 (20.8%)	935 (22.0%)	1.16%	1.07 (0.98–1.17)	0.14
LOS in ICU, days (median (IQR))	3.7 (1.9–7.4)	3.6 (1.7–7.5)	−0.1	–	0.42
LOS in ICU for survivors (median (IQR))	3.8 (1.9–7.3)	3.7 (1.8–7.6)	−0.1	–	0.68
LOS in ICU for non-survivors (median (IQR))	2.6 (0.9–7.6)	2.4 (0.7–6.1)	−0.2	–	0.039
LOS in hospital, days (median (IQR))	14 (6–30)	13 (5–28)	−1.0	–	0.0006

*CI* confidence interval, *ICU* intensive care unit, *IQR* interquartile range, *LOS* length of stay, *OR* odds ratio

**Table 4:** Summary of direct, indirect, and total (integrated) effect of afterhours admission on ICU mortality, hospital mortality, and lengths of stay

Outcome	Direct effect		Indirect effect		Total (integrated effect)	
	OR (95% CI)	*p* value	OR (95% CI)	*p* value	OR (95% CI)	*p* value
Mortality						
Death in ICU	0.96 (0.87–1.06)	0.39	1.07 (1.05–1.09)	<.0001	1.02 (0.92–1.14)	0.66
Death within 30 h in ICU	1.00 (0.83–1.22)	0.97	1.11 (1.07–1.14)	<.0001	1.11 (0.91–1.35)	0.30
Death within 3 days in ICU	0.97 (0.83–1.14)	0.74	1.09 (1.06–1.12)	<.0001	1.06 (0.91–1.24)	0.46
Death within 7 days in ICU	1.01 (0.88–1.15)	0.94	1.08 (1.06–1.11)	<.0001	1.09 (0.95–1.24)	0.21
Death in hospital	0.94 (0.86–1.03)	0.21	1.05 (1.04–1.07)	<.0001	0.99 (0.91–1.09)	0.89
Length of stay						
LOS in ICU	0.93 (0.88–0.98)	0.013	1.02 (1.01–1.02)	<.0001	0.94 (0.89–1.00)	0.0498
LOS in ICU for survivors	0.94 (0.90–0.98)	0.0024	1.03 (1.02–1.04)	<.0001	0.97 (0.93–1.01)	0.11
LOS in ICU for nonsurvivors	1.02 (0.85–1.21)	0.86	0.97 (0.96–0.98)	<.0001	0.98 (0.83–1.17)	0.86
LOS in hospital	0.96 (0.90–1.02)	0.20	1.01 (1.00–1.01)	0.021	0.96 (0.90–1.03)	0.27

OR estimates and their 95% CIs were calculated based on parameter estimates; parameter estimates of integrated effect were based on 1 million times simulation experiments

*CI* confidence interval, *ICU* intensive care unit, *LOS* length of stay, *OR* odds ratio

Afterhours admissions had worse short-term outcomes in the presence of indicators of strained ICU capacity. For admissions occurring between 00:00 and 06:59 h and while bed occupancy was ≥ 90%, ICU mortality was greater. Similarly, for afterhours admission occurring during relative clustering of ICU admissions, short-term ICU mortality (< 7 days) was higher (Table 5).

**Table 5:** Sensitivity analysis demonstrating the integrated effect of various admission times and admission conditions on ICU mortality, hospital mortality, and length of stay

Outcome	Between 00:00 and 06:59h		Between 00:00 and 06:59 h on non-work day		Between 00:00 and 06:59 h and during October–March	
	OR (95% CI)	*p* value	OR (95% CI)	*p* value	OR (95% CI)	*p* value
Mortality						
Death in ICU	1.10 (0.98–1.23)	0.10	1.13 (0.96–1.34)	0.14	1.15 (0.99–1.33)	0.060
Death within 30 h in ICU	1.28 (1.03–1.58)	0.024	1.44 (1.07–1.94)	0.018	1.38 (1.06–1.80)	0.016
Death within 3 days in ICU	1.17 (0.98–1.39)	0.075	1.34 (1.04–1.72)	0.023	1.37 (1.11–1.70)	0.0039
Death within 7 days in ICU	1.14 (0.98–1.31)	0.091	1.34 (1.09–1.66)	0.0068	1.23 (1.02–1.49)	0.028
Death in hospital	1.02 (0.92–1.13)	0.70	1.08 (0.93–1.26)	0.33	1.13 (0.99–1.29)	0.065
Length of stay						
LOS in ICU	0.80 (0.66–0.97)	0.026	0.80 (0.67–0.94)	0.0084	0.89 (0.72–1.09)	0.26
LOS in hospital	1.01 (0.85–1.20)	0.91	0.94 (0.81–1.10)	0.43	0.98 (0.82–1.16)	0.78
	Between 00:00 and 06:59 h and bed occupancy ≥ 90%		Between 00:00 and 06:59 h and bed occupancy ≥ 95%		Afterhours and clustering admissions per bed ≥ 0.15	
	OR (95% CI)	*p* value	OR (95% CI)	*p* value	OR (95% CI)	*p* value
Mortality						
Death in ICU	1.21 (1.03–1.43)	0.019	1.26 (1.01–1.58)	0.039	1.08 (0.97–1.20)	0.15
Death within 30 h in ICU	1.37 (1.02–1.85)	0.037	1.34 (0.88–2.03)	0.18	1.25 (1.02–1.53)	0.030
Death within 3 days in ICU	1.33 (1.04–1.69)	0.021	1.26 (0.91–1.76)	0.17	1.18 (1.00–1.38)	0.049
Death within 7 days in ICU	1.25 (1.01–1.54)	0.039	1.24 (0.93–1.66)	0.15	1.16 (1.01–1.33)	0.034
Death in hospital	1.11 (0.96–1.29)	0.17	1.12 (0.91–1.37)	0.28	1.05 (0.95–1.15)	0.33
Length of stay						
LOS in ICU	0.79 (0.63–0.98)	0.031	0.97 (0.91–1.03)	0.26	0.94 (0.89–1.01)	0.083
LOS in hospital	1.08 (0.88–1.33)	0.46	1.02 (0.90–1.16)	0.72	0.97 (0.90–1.04)	0.32

OR estimates and their 95% CIs were calculated based on parameter estimates; parameter estimates of integrated effect were based on 1 million times simulation experiments

*CI* confidence interval, *ICU* intensive care unit, *LOS* length of stay, *OR* odds ratio

### Association between afterhours admission and secondary outcomes

Hospital mortality was 21.3% (*n* = 2605) with no significant difference between afterhours and daytime ICU admission in unadjusted, direct, or integrated (mediated through APACHE II score) analyses (Tables 3 and 4). In multivariate analysis, afterhours compared with daytime ICU admission was associated with reduced ICU LOS in direct and integrated analysis; however, this was not significant when stratified by ICU survival status. There was no association with afterhours admission and hospital LOS.

### Sensitivity analyses

Early morning admission occurring between 00:00 and 06:59 h, between 00:00 and 06:59 h on weekend/holidays, and between 00:00 and 06:59 h during winter months showed association with early death in the ICU (death within 30 h in the ICU) and shorter ICU LOS, likely driven by ICU survivors (Tables 4 and 5); however, admissions during these periods were not associated with total ICU or hospital mortality, or with hospital LOS (Table 5).

## Discussion

In this multicenter population-based cohort study in a large Canadian health region, we found that approximately one-third of all ICU admissions occurred afterhours. The proportion of afterhours admissions varied across sites, with a greater proportion occurring in academic hospital ICUs. Patients admitted afterhours were younger, had greater illness acuity, were more likely medical (non-operative), and were most commonly referred from the emergency department. We found no effect of afterhours admission on ICU or hospital mortality (either direct or after integrating effects mediated through illness severity); however, we showed that this effect may be sensitive to whether an ICU is experiencing strained capacity. Afterhours admission during periods of high occupancy was associated with increases in early ICU mortality and shorter ICU LOS, while concomitant clustering of admissions also showed association with early ICU mortality. Afterhours admission, including during periods of strain, was not, however, associated with greater all-cause hospital mortality.

### Interpretation with prior work

Afterhours ICU admissions have shown inconsistent association with adverse patient outcomes when compared to admissions occurring during usual daytime hours. This variability is likely attributable to differences in study design, to varying definitions for “afterhours”, heterogeneous case mix, differential risk of bias and residual confounding, type of health jurisdiction being evaluated, and, importantly, due to likely small estimates for differences in effect [20, 21]. Two systematic reviews have suggested no incremental hazard for afterhours ICU admission and mortality risk; however, both suggested greater risk for admissions occurring on weekends relative to weekdays, and further suggested estimates may be sensitive to ICU organizational structure and geographic variation [20, 21]. In a large multicenter cohort study from Australia (*n* = 245,057), afterhours (18:00–05:59 h) and weekend admissions were shown to have greater risk-adjusted hospital mortality compared with daytime admissions [27]. While mortality was marginally higher in our cohort and largely mediated by illness severity, our study adds new knowledge by suggesting that system-level variables such as strained capacity (i.e., high occupancy) and workload (i.e., clustering of ICU admissions), when coupled with admissions occurring afterhours, may further exert small but important effects on patient risk of adverse outcome.

A spectrum of system-related factors may account for the purported increased mortality risk associated with afterhours ICU admissions. ICU organizational structure, including reduced afterhours intensivist coverage and nurse-to-patient ratios, may negatively impact ICU care afterhours, particularly during strained capacity conditions [28]. Adoption of an in-house intensivist staffing model has shown variable effect to improve care processes and outcomes; however, no study has specifically evaluated how this coverage model performs during strain [29–34]. Similarly, greater bedside nursing workload may compromise care quality and increase the risk of adverse outcomes [35]; however, no prior work has evaluated the relationship between afterhours admission and workload during strained conditions. Nursing workload may be susceptible to both patient-level factors (i.e., acuity) and system-level factors (i.e., reduced nurse-to-patient ratios afterhours); and these relationships may be further exploited when ICUs become strained, such as during a large number of new admissions occurring within a relatively short period of time [36, 37].

### Implications for practice, research, and policy

Our study supports the notion that both patient-specific and system-related factors may contribute to a small but important mortality risk associated with afterhours ICU admissions. While our work should be further replicated, it would imply at a minimum that individual ICUs and/or health regions should interrogate data on afterhours admissions, particularly during periods of strain. Future work should also examine for system-related patterns of strained ICU capacity that may be foreseeable, that may be further targets of quality improvement initiatives, and which could inform capacity and workforce planning, and policy development aimed at mitigating avoidable risk.

The greater acuity for afterhours admissions could be attributed to several factors, including patients whose ICU transfer was delayed or who received suboptimal initial resuscitation, more unplanned admissions (i.e., emergency surgery), or admission of sicker patients with reduced likelihood of deriving benefit from ICU care (i.e., futile ICU admission nearing end of life), due in part to different staffing models (i.e., no in-house intensivist coverage afterhours) [38]. These same factors may account for the higher early mortality occurring among afterhours admissions during periods of strain. The greater rates of afterhours admissions at academic sites in our study would support these hypotheses. Alternatively, Bhonagiri et al. [27] found greater mortality for afterhours admissions among elective surgery patients. This was speculated to occur due to delays in ICU admission from complicated intraoperative courses that were not otherwise captured in illness severity scores. In our cohort, however, elective surgical patients represented only 9.9% of ICU admissions and were consistently associated with reduced mortality in multivariate analysis [39].

### Limitations

While our study was a relatively large multicenter population-based interrogation of routinely captured ICU clinical and administrative data, our study has limitations that warrant consideration. First, despite extensive covariate adjustment, our study is observational and remains susceptible to bias and residual confounding. Second, our cohort was restricted to those admitted to the ICU. We did not have data on patients who were referred to or received ICU consultation but were declined admission or those whose ICU admission was potentially delayed [40, 41]. Similarly, we did not have data on patient goals of care at the time of ICU admission or changes that occurred thereafter, recognizing their potential interaction with strained capacity [42]. Third, we defined afterhours admission as occurring between 22:00 and 06:59 h to largely reflect contemporaneous practice in our health region; and while we applied a variety of sensitivity analyses to this definition, we recognize this may not be generalizable to other jurisdictions with variable ICU organization structures. Fourth, our study focused on relatively proximate patient outcomes occurring in the ICU and hospital; as such, we cannot comment on alternative outcomes that may be associated with afterhours ICU admission including major morbidity, satisfaction with care, and long-term mortality.

## Conclusions

Afterhours ICU admission is common and associated with increased illness severity. Although afterhours ICU admission did not portend greater mortality risk in most circumstances, this association may be sensitive to strained ICU capacity conditions. Future work should focus on evaluating those modifiable factors, particularly if related to ICU organizational structure that may mediate mortality risk associated with afterhours ICU admission.

## Additional files

Additional file 1: Summary of path-analysis modeling strategy. (DOCX 33 kb)

Additional file 2: Multivariate, mixed-effects linear regression of factors associated with admission APACHE II score. (DOCX 20 kb)

Additional file 3: Multivariate, mixed-effects logistic regression on ICU mortality within 30 h. (DOCX 21 kb)

Additional file 4: Multivariate, mixed-effects logistic regression on ICU mortality within 3 days. (DOCX 21 kb)

Additional file 5: Multivariate, mixed-effects logistic regression on ICU mortality within 7 days. (DOCX 21 kb)

Additional file 6: Multivariate, mixed-effects logistic regression on ICU mortality. (DOCX 22 kb)

Additional file 7: Multivariate, mixed effects logistic regression on hospital mortality. (DOCX 21 kb)

Additional file 8: Direct effects of admission APACHE II score on ICU mortality, hospital mortality, and length of stay. (DOCX 18 kb)

Additional file 9: Multivariate, mixed-effects Poisson regression model for hospital length of stay. (DOCX 20 kb)

## Abbreviations

AIDS: Acute immunodeficiency syndrome
APACHE II: Acute Physiology and Chronic Health Evaluation II
CI: Confidence interval
ED: Emergency department
ICU: Intensive care unit
IQR: Interquartile range
LOS: Length of stay
OR: Odds ratio
SD: Standard deviation
SE: Standard error
