# Supplementary material for: Association between afterhours admission to the intensive care unit, strained capacity, and mortality: a retrospective cohort study
Source: Crit Care. 2018 Apr 17;22:97. doi: 10.1186/s13054-018-2027-8 (PMC5905119; doi:10.1186/s13054-018-2027-8)
Supplement: Supplementary file 1 — Summary of path-analysis modeling strategy. (DOCX 33 kb) [file 13054_2018_2027_MOESM1_ESM.docx]

**Additional File 1.** Summary of path-analysis modelling strategy.

$$e_{1}\sim N(E_{1}, S_{1})$$

**Afterhours ICU Admission**

**ICU Mortality**

**Admission APACHE II Score**

$$e_{3}\sim N(E_{3}, S_{3})$$

$$e_{2}\sim N(E_{2}, S_{2})$$

The simulation process for ICU mortality was done using R package and this diagram. The simulation process for ICU mortality within 30 hours, 3 days or 7 days as well as hospital mortality was done similarly.

After modeling, we extracted three pairs of parameters $\left( E_{i}, S_{i} \right), i=1, 2, 3$ (estimate, standard error), which were used to describe the direct and indirect associations from after-hour admission on ICU mortality. In detail, we used normal distribution $N(E_{1}, S_{1})$ to describe the direct association between afterhours ICU admission and ICU mortality, and $N\left( E_{2}, S_{2} \right)$and $N\left( E_{3}, S_{3} \right)$ to describe the indirect association. We did a simulation with 1 million replications to calculate integrated effect using the following algorithm:

Algorithm for estimating the integrated effect of afterhours ICU admission.

*Initialize:* Assign$\left( E_{i}, S_{i} \right), i=1, 2, 3$.

*Iterate:* Repeat step 1-2 one million times

Step 1 created three random numbers from the three normal distributions, i.e. $e_{i} \sim N\left( E_{i}, S_{i} \right), i = 1, 2, 3$.

Step 2 calculate indirect effect and integrated effect

$$Indirect Effect=e_{2}*e_{3}.$$

$$Integrated Effect=e_{1}+e_{2}*e_{3}.$$

*Summarize:* calculate coefficient estimate (mean), SE (standard error), p-value (two sided), OR (odds ratio) and 95% CI (confident interval) of indirect/integrated effect from simulated one million indirect/integrated effect values.
