# Supplementary material for: Association between afterhours admission to the intensive care unit, strained capacity, and mortality: a retrospective cohort study
Source: Crit Care. 2018 Apr 17;22:97. doi: 10.1186/s13054-018-2027-8 (PMC5905119; doi:10.1186/s13054-018-2027-8)
Supplement: Supplementary file 2 — Multivariate, mixed-effects linear regression of factors associated with admission APACHE II score. (DOCX 20 kb) [file 13054_2018_2027_MOESM2_ESM.docx]

**Additional File 2.** Multivariate, mixed effects linear regression of factors associated with admission APACHE II score.

| **Effect** | **Estimate** | **SE** | **p-value** |
| --- | --- | --- | --- |
| **Intercept** | 11.5850 | 0.6508 | <.0001 |
| **Age** |  |  |  |
| < 65 years | reference |  |  |
| 65-74 years | 2.9606 | 0.1622 | <.0001 |
| 75-84 years | 3.6525 | 0.1876 | <.0001 |
| ≥ 85 years | 3.5580 | 0.3408 | <.0001 |
| **Sex** |  |  |  |
| Female | reference |  |  |
| Male | -0.3076 | 0.1237 | 0.0129 |
| **Location** |  |  |  |
| Calgary | reference |  |  |
| Edmonton | 1.0905 | 0.6854 | 0.1117 |
| **System** |  |  |  |
| Cardiovascular | reference |  |  |
| Gastrointestinal | -1.4609 | 0.2538 | <.0001 |
| Genitourinary | -1.9976 | 0.3478 | <.0001 |
| Hematology | -2.6827 | 0.8283 | 0.0012 |
| Metabolic/Endocrine | -1.1887 | 0.4500 | 0.0083 |
| Musculoskeletal/Skin | -1.6912 | 0.3565 | <.0001 |
| Neurologic | -2.5894 | 0.2597 | <.0001 |
| Respiratory | -1.3488 | 0.1979 | <.0001 |
| Transplant | -4.5778 | 0.6377 | <.0001 |
| Trauma | -2.1520 | 0.4103 | <.0001 |
| **Surgery** |  |  |  |
| Non-operative | reference |  |  |
| Elective | -1.2585 | 0.3135 | <.0001 |
| Emergent | 0.2920 | 0.2631 | 0.2670 |
| **Class** |  |  |  |
| Medical | reference |  |  |
| Neurological | 1.2016 | 0.3012 | <.0001 |
| Surgical | -0.3020 | 0.2714 | 0.2659 |
| Trauma without head injury | -0.5974 | 0.4406 | 0.1751 |
| Trauma with head injury | 2.1060 | 0.4956 | <.0001 |
| **Comorbidity** |  |  |  |
| Chronic Dialysis | 5.0874 | 0.3422 | <.0001 |
| Hepatic | 1.5772 | 0.2415 | <.0001 |
| Neurologic | 4.5240 | 0.1468 | <.0001 |
| AIDS | 2.0175 | 0.8318 | 0.0153 |
| Chronic Heart | 1.9030 | 0.2667 | <.0001 |
| Respiratory | 3.5019 | 0.2020 | <.0001 |
| Metastatic/ Leukemia/ Lymphoma | 0.9196 | 0.2649 | 0.0005 |
| Immune Suppression | 3.5914 | 0.2251 | <.0001 |
| Diabetes | 0.3374 | 0.1707 | 0.0481 |
| Cirrhosis | 3.2404 | 0.2997 | <.0001 |
| Cardiovascular | 2.0469 | 0.1416 | <.0001 |
| Digestive | 0.7066 | 0.1995 | 0.0004 |
| Acute Renal | 5.6746 | 0.1580 | <.0001 |
| **Occupancy** | 0.0227 | 0.0046 | <.0001 |
| **Charlson Comorbidity Index** | 0.4778 | 0.0620 | <.0001 |
| **Afterhours admission** | 0.7763 | 0.1279 | <.0001 |
| *Definition of abbreviation*: SE=standard error.  Stepwise variable selection procedure was adopted to eliminate one-by-one those variables (other than the main exposure variable) with p-value over 0.25. | | | |
