# Supplementary material for: Association between afterhours admission to the intensive care unit, strained capacity, and mortality: a retrospective cohort study
Source: Crit Care. 2018 Apr 17;22:97. doi: 10.1186/s13054-018-2027-8 (PMC5905119; doi:10.1186/s13054-018-2027-8)
Supplement: Supplementary file 3 — Multivariate, mixed-effects logistic regression on ICU mortality within 30 h. (DOCX 21 kb) [file 13054_2018_2027_MOESM3_ESM.docx]

**Additional File 3.** Multivariate, mixed effects logistic regression of ICU mortality within 30 hours.

| **Effect** | **Estimate** | **SE** | **p-value** | **OR 95% CI** | | |
| --- | --- | --- | --- | --- | --- | --- |
|  |  |  |  | **OR** | **LCL** | **UCL** |
| **Intercept** | -14.5869 | 0.4441 | <.0001 |  |  |  |
| **Age** |  |  |  |  |  |  |
| < 65 years | reference |  |  |  |  |  |
| 65-74 years | 0.03976 | 0.1242 | 0.7489 | 1.041 | 0.816 | 1.327 |
| 75-84 years | 0.1921 | 0.1367 | 0.1601 | 1.212 | 0.927 | 1.584 |
| ≥ 85 years | 0.6462 | 0.1991 | 0.0012 | 1.908 | 1.292 | 2.819 |
| **Sex** |  |  |  |  |  |  |
| Female | reference |  |  |  |  |  |
| Male | -0.2346 | 0.0989 | 0.0177 | 0.791 | 0.652 | 0.960 |
| **Hospital type** |  |  |  |  |  |  |
| Academic | reference |  |  |  |  |  |
| Community | 1.1339 | 0.4698 | 0.0678 | 3.108 | 1.238 | 7.804 |
| Tertiary | 0.4327 | 0.5306 | 0.4638 | 1.541 | 0.545 | 4.361 |
| **System** |  |  |  |  |  |  |
| Cardiovascular | reference |  |  |  |  |  |
| Gastrointestinal | -0.1715 | 0.1608 | 0.2861 | 0.842 | 0.615 | 1.154 |
| Genitourinary | -0.9390 | 0.3904 | 0.0162 | 0.391 | 0.182 | 0.840 |
| Hematology | -0.0762 | 0.7227 | 0.9160 | 0.927 | 0.225 | 3.820 |
| Metabolic/Endocrine | -1.5277 | 0.7138 | 0.0324 | 0.217 | 0.054 | 0.879 |
| Musculoskeletal/Skin | -0.5267 | 0.3690 | 0.1535 | 0.591 | 0.287 | 1.217 |
| Neurologic | -0.7343 | 0.2251 | 0.0011 | 0.480 | 0.309 | 0.746 |
| Respiratory | -0.6170 | 0.1537 | <.0001 | 0.540 | 0.399 | 0.729 |
| Transplant | -0.4942 | 1.0288 | 0.6310 | 0.610 | 0.081 | 4.582 |
| Trauma | -0.5920 | 0.3032 | 0.0509 | 0.553 | 0.305 | 1.002 |
| **Class** |  |  |  |  |  |  |
| Medical | reference |  |  |  |  |  |
| Neurological | 1.0654 | 0.2109 | <.0001 | 2.902 | 1.919 | 4.387 |
| Surgical | -0.3107 | 0.1643 | 0.0586 | 0.733 | 0.531 | 1.011 |
| Trauma without head injury | -0.0985 | 0.4535 | 0.8280 | 0.906 | 0.373 | 2.204 |
| Trauma with head injury | 0.9070 | 0.2993 | 0.0024 | 2.477 | 1.378 | 4.453 |
| **Comorbidity** |  |  |  |  |  |  |
| Chronic Dialysis | -0.8630 | 0.2879 | 0.0027 | 0.422 | 0.240 | 0.742 |
| Hepatic | 0.1782 | 0.1304 | 0.1718 | 1.195 | 0.926 | 1.543 |
| Chronic Heart | -0.2403 | 0.1678 | 0.1520 | 0.786 | 0.566 | 1.093 |
| Metastatic/ Leukemia/ Lymphoma | 0.5046 | 0.1733 | 0.0036 | 1.656 | 1.179 | 2.326 |
| Immune Suppression | -0.7319 | 0.1861 | <.0001 | 0.481 | 0.334 | 0.693 |
| Cardiovascular | 0.5078 | 0.1397 | 0.0003 | 1.662 | 1.264 | 2.185 |
| Digestive | 0.4097 | 0.1327 | 0.0020 | 1.506 | 1.161 | 1.954 |
| **Admission APACHE II score** | 0.1289 | 0.0062 | <.0001 | 1.138 | 1.124 | 1.151 |
| **Afterhours admission** | 0.0037 | 0.0998 | 0.9703 | 1.004 | 0.825 | 1.220 |
| *Definition of abbreviation*: SE=standard error; CI=confident interval.  Stepwise variable selection procedure was adopted to eliminate one-by-one those variables (other than the main exposure variable) with p-value over 0.25. | | | | | | |
