# Supplementary material for: Association between afterhours admission to the intensive care unit, strained capacity, and mortality: a retrospective cohort study
Source: Crit Care. 2018 Apr 17;22:97. doi: 10.1186/s13054-018-2027-8 (PMC5905119; doi:10.1186/s13054-018-2027-8)
Supplement: Supplementary file 4 — Multivariate, mixed-effects logistic regression on ICU mortality within 3 days. (DOCX 21 kb) [file 13054_2018_2027_MOESM4_ESM.docx]

**Additional File 4.** Multivariate, mixed effects logistic regression of ICU mortality within 3 days.

| **Effect** | **Estimate** | **SE** | **p-value** | **OR 95% CI** | | |
| --- | --- | --- | --- | --- | --- | --- |
|  |  |  |  | **OR** | **LCL** | **UCL** |
| **Intercept** | -13.5951 | 0.4227 | <.0001 |  |  |  |
| **Age** |  |  |  |  |  |  |
| < 65 years | reference |  |  |  |  |  |
| 65-74 years | 0.1049 | 0.0975 | 0.2819 | 1.111 | 0.918 | 1.344 |
| 75-84 years | 0.1689 | 0.1093 | 0.1222 | 1.184 | 0.956 | 1.467 |
| ≥ 85 years | 0.7659 | 0.1534 | <.0001 | 2.151 | 1.592 | 2.905 |
| **Hospital type** |  |  |  |  |  |  |
| Academic | reference |  |  |  |  |  |
| Community | 1.4888 | 0.4615 | 0.0197 | 4.432 | 1.794 | 10.950 |
| Tertiary | 0.3990 | 0.5407 | 0.4923 | 1.490 | 0.516 | 4.301 |
| **System** |  |  |  |  |  |  |
| Cardiovascular | reference |  |  |  |  |  |
| Gastrointestinal | -0.2490 | 0.1318 | 0.0588 | 0.780 | 0.602 | 1.009 |
| Genitourinary | -0.8068 | 0.2779 | 0.0037 | 0.446 | 0.259 | 0.769 |
| Hematology | -0.2844 | 0.5882 | 0.6287 | 0.752 | 0.238 | 2.383 |
| Metabolic/Endocrine | -1.1053 | 0.4540 | 0.0149 | 0.331 | 0.136 | 0.806 |
| Musculoskeletal/Skin | -0.5241 | 0.2715 | 0.0536 | 0.592 | 0.348 | 1.008 |
| Neurologic | -0.6708 | 0.1741 | 0.0001 | 0.511 | 0.363 | 0.719 |
| Respiratory | -0.4959 | 0.1139 | <.0001 | 0.609 | 0.487 | 0.761 |
| Transplant | -1.5525 | 1.0186 | 0.1275 | 0.212 | 0.029 | 1.559 |
| Trauma | -0.6167 | 0.2568 | 0.0163 | 0.540 | 0.326 | 0.893 |
| **Surgery** |  |  |  |  |  |  |
| Non-operative | reference |  |  |  |  |  |
| Elective | -0.6918 | 0.3564 | 0.0523 | 0.501 | 0.249 | 1.007 |
| Emergent | 0.0740 | 0.1853 | 0.6896 | 1.077 | 0.749 | 1.548 |
| **Class** |  |  |  |  |  |  |
| Medical | reference |  |  |  |  |  |
| Neurological | 1.1224 | 0.1655 | <.0001 | 3.072 | 2.221 | 4.249 |
| Surgical | -0.1915 | 0.1901 | 0.3137 | 0.826 | 0.569 | 1.199 |
| Trauma without head injury | -0.4203 | 0.3922 | 0.2840 | 0.657 | 0.305 | 1.417 |
| Trauma with head injury | 0.8121 | 0.2609 | 0.0019 | 2.253 | 1.351 | 3.756 |
| **Comorbidity** |  |  |  |  |  |  |
| Hepatic | 0.4520 | 0.1013 | <.0001 | 1.571 | 1.288 | 1.917 |
| Chronic Dialysis | -0.3740 | 0.1909 | 0.0501 | 0.688 | 0.473 | 1.000 |
| Metastatic/ Leukemia/ Lymphoma | 0.2960 | 0.1335 | 0.0266 | 1.344 | 1.035 | 1.747 |
| Cardiovascular | 0.5140 | 0.1085 | <.0001 | 1.672 | 1.352 | 2.068 |
| Digestive | 0.1833 | 0.1082 | 0.0902 | 1.201 | 0.972 | 1.485 |
| **Admission APACHE II score** | 0.1106 | 0.0047 | <.0001 | 1.117 | 1.107 | 1.127 |
| **Charlson Index** | -0.0788 | 0.0345 | 0.0223 | 0.924 | 0.864 | 0.989 |
| **Afterhours admission** | -0.0260 | 0.0795 | 0.7440 | 0.974 | 0.834 | 1.139 |
| *Definition of abbreviation*: SE=standard error; CI=confident interval.  Stepwise variable selection procedure was adopted to eliminate one-by-one those variables (other than the main exposure variable) with p-value over 0.25. | | | | | | |
