# Supplementary material for: Association between afterhours admission to the intensive care unit, strained capacity, and mortality: a retrospective cohort study
Source: Crit Care. 2018 Apr 17;22:97. doi: 10.1186/s13054-018-2027-8 (PMC5905119; doi:10.1186/s13054-018-2027-8)
Supplement: Supplementary file 5 — Multivariate, mixed-effects logistic regression on ICU mortality within 7 days. (DOCX 21 kb) [file 13054_2018_2027_MOESM5_ESM.docx]

**Additional File 5.** Multivariate, mixed effects logistic regression of ICU mortality within 7 days.

| **Effect** | **Estimate** | **SE** | **p-value** | **OR 95% CI** | | |
| --- | --- | --- | --- | --- | --- | --- |
|  |  |  |  | **OR** | **LCL** | **UCL** |
| **Intercept** | -12.8176 | 0.4048 | <.0001 |  |  |  |
| **Age** |  |  |  |  |  |  |
| < 65 years | reference |  |  |  |  |  |
| 65-74 years | 0.0130 | 0.0844 | 0.8775 | 1.013 | 0.859 | 1.195 |
| 75-84 years | 0.1809 | 0.0913 | 0.0475 | 1.198 | 1.002 | 1.433 |
| ≥ 85 years | 0.6908 | 0.1302 | <.0001 | 1.995 | 1.546 | 2.575 |
| **Hospital type** |  |  |  |  |  |  |
| Academic | reference |  |  |  |  |  |
| Community | 1.5846 | 0.4516 | 0.0137 | 4.877 | 2.013 | 11.819 |
| Tertiary | 0.3712 | 0.5329 | 0.5147 | 1.449 | 0.510 | 4.119 |
| **System** |  |  |  |  |  |  |
| Cardiovascular | reference |  |  |  |  |  |
| Gastrointestinal | -0.1790 | 0.1038 | 0.0846 | 0.836 | 0.682 | 1.025 |
| Genitourinary | -0.9592 | 0.2511 | 0.0001 | 0.383 | 0.234 | 0.627 |
| Hematology | -0.2089 | 0.4575 | 0.6479 | 0.811 | 0.331 | 1.989 |
| Metabolic/Endocrine | -1.2037 | 0.3838 | 0.0017 | 0.300 | 0.141 | 0.637 |
| Musculoskeletal/Skin | -0.5382 | 0.2295 | 0.0190 | 0.584 | 0.372 | 0.915 |
| Neurologic | -0.8401 | 0.1477 | <.0001 | 0.432 | 0.323 | 0.577 |
| Respiratory | -0.4001 | 0.0942 | <.0001 | 0.670 | 0.557 | 0.806 |
| Transplant | -1.7215 | 1.0142 | 0.0897 | 0.179 | 0.024 | 1.305 |
| Trauma | -0.9009 | 0.2184 | <.0001 | 0.406 | 0.265 | 0.623 |
| **Surgery** |  |  |  |  |  |  |
| Non-operative | reference |  |  |  |  |  |
| Elective | -0.8489 | 0.3120 | 0.0065 | 0.428 | 0.232 | 0.789 |
| Emergent | 0.1603 | 0.1588 | 0.3130 | 1.174 | 0.860 | 1.602 |
| **Class** |  |  |  |  |  |  |
| Medical | reference |  |  |  |  |  |
| Neurological | 1.1149 | 0.1391 | <.0001 | 3.049 | 2.322 | 4.005 |
| Surgical | -0.2986 | 0.1646 | 0.0697 | 0.742 | 0.537 | 1.024 |
| Trauma without head injury | -0.5569 | 0.3585 | 0.1203 | 0.573 | 0.284 | 1.157 |
| Trauma with head injury | 0.9390 | 0.2157 | <.0001 | 2.557 | 1.676 | 3.903 |
| **Comorbidity** |  |  |  |  |  |  |
| Chronic Dialysis | -0.4211 | 0.1690 | 0.0127 | 0.656 | 0.471 | 0.914 |
| Hepatic | 0.4009 | 0.0877 | <.0001 | 1.493 | 1.257 | 1.773 |
| Metastatic/ Leukemia/ Lymphoma | 0.4030 | 0.1206 | 0.0008 | 1.496 | 1.181 | 1.895 |
| Immune Suppression | -0.3396 | 0.1150 | 0.0032 | 0.712 | 0.568 | 0.892 |
| Cardiovascular | 0.3508 | 0.0868 | <.0001 | 1.420 | 1.198 | 1.684 |
| **Admission APACHE II score** | 0.1025 | 0.0041 | <.0001 | 1.108 | 1.099 | 1.117 |
| **Charlson Index** | -0.0598 | 0.0289 | 0.0384 | 0.942 | 0.890 | 0.997 |
| **Afterhours admission** | 0.0052 | 0.0670 | 0.9381 | 1.005 | 0.881 | 1.146 |
| *Definition of abbreviation*: SE=standard error; CI=confident interval.  Stepwise variable selection procedure was adopted to eliminate one-by-one those variables (other than the main exposure variable) with p-value over 0.25. | | | | | | |
