# Supplementary material for: Association between afterhours admission to the intensive care unit, strained capacity, and mortality: a retrospective cohort study
Source: Crit Care. 2018 Apr 17;22:97. doi: 10.1186/s13054-018-2027-8 (PMC5905119; doi:10.1186/s13054-018-2027-8)
Supplement: Supplementary file 6 — Multivariate, mixed-effects logistic regression on ICU mortality. (DOCX 22 kb) [file 13054_2018_2027_MOESM6_ESM.docx]

**Additional File 6.** Multivariate, mixed effects logistic regression of ICU mortality.

| **Effect** | **Estimate** | **SE** | **p-value** | **OR 95% CI** | | |
| --- | --- | --- | --- | --- | --- | --- |
|  |  |  |  | **OR** | **LCL** | **UCL** |
| **Intercept** | -12.0903 | 0.3772 | <.0001 |  |  |  |
| **Age** |  |  |  |  |  |  |
| < 65 years | reference |  |  |  |  |  |
| 65-74 years | 0.0945 | 0.0630 | 0.1333 | 1.099 | 0.972 | 1.243 |
| 75-84 years | 0.1677 | 0.0707 | 0.0177 | 1.183 | 1.030 | 1.358 |
| ≥ 85 years | 0.6915 | 0.1032 | <.0001 | 1.997 | 1.631 | 2.444 |
| **Sex** |  |  |  |  |  |  |
| Female | reference |  |  |  |  |  |
| Male | -0.0682 | 0.0509 | 0.1801 | 0.934 | 0.845 | 1.032 |
| **Hospital type** |  |  |  |  |  |  |
| Academic | reference |  |  |  |  |  |
| Community | 1.6328 | 0.4300 | 0.0096 | 5.118 | 2.203 | 11.889 |
| Tertiary | 0.4794 | 0.5089 | 0.3850 | 1.615 | 0.596 | 4.379 |
| **System** |  |  |  |  |  |  |
| Cardiovascular | reference |  |  |  |  |  |
| Gastrointestinal | -0.1449 | 0.0896 | 0.1059 | 0.865 | 0.726 | 1.031 |
| Genitourinary | -0.9297 | 0.2109 | <.0001 | 0.395 | 0.261 | 0.597 |
| Hematology | 0.2781 | 0.3109 | 0.3711 | 1.321 | 0.718 | 2.429 |
| Metabolic/Endocrine | -1.4446 | 0.3577 | <.0001 | 0.236 | 0.117 | 0.475 |
| Musculoskeletal/Skin | -0.3158 | 0.1719 | 0.0662 | 0.729 | 0.521 | 1.021 |
| Neurologic | -0.7380 | 0.1145 | <.0001 | 0.478 | 0.382 | 0.598 |
| Respiratory | -0.0963 | 0.0697 | 0.1673 | 0.908 | 0.792 | 1.041 |
| Transplant | -1.0295 | 0.5908 | 0.0814 | 0.357 | 0.112 | 1.137 |
| Trauma | -0.6446 | 0.1649 | <.0001 | 0.525 | 0.380 | 0.725 |
| **Surgery** |  |  |  |  |  |  |
| Non-operative | reference |  |  |  |  |  |
| Elective | -1.0912 | 0.2305 | <.0001 | 0.336 | 0.214 | 0.528 |
| Emergent | -0.1330 | 0.1176 | 0.2582 | 0.875 | 0.695 | 1.102 |
| **Class** |  |  |  |  |  |  |
| Medical | reference |  |  |  |  |  |
| Neurological | 0.9547 | 0.1112 | <.0001 | 2.598 | 2.089 | 3.231 |
| Surgical | -0.0944 | 0.1199 | 0.4312 | 0.910 | 0.719 | 1.151 |
| Trauma without head injury | 0.1029 | 0.2148 | 0.6318 | 1.108 | 0.728 | 1.689 |
| Trauma with head injury | 0.8622 | 0.1695 | <.0001 | 2.368 | 1.699 | 3.302 |
| **Comorbidity** |  |  |  |  |  |  |
| Chronic Dialysis | -0.2822 | 0.1284 | 0.0279 | 0.754 | 0.586 | 0.970 |
| Hepatic | 0.4045 | 0.0689 | <.0001 | 1.499 | 1.309 | 1.715 |
| Metastatic/ Leukemia/ Lymphoma | 0.2416 | 0.0881 | 0.0061 | 1.273 | 1.071 | 1.513 |
| Cardiovascular | 0.2325 | 0.0632 | 0.0002 | 1.262 | 1.115 | 1.428 |
| Digestive | 0.1045 | 0.0729 | 0.1518 | 1.110 | 0.962 | 1.281 |
| **Admission APACHE II score** | 0.0867 | 0.0031 | <.0001 | 1.091 | 1.084 | 1.097 |
| **Afterhours admission** | -0.0446 | 0.0517 | 0.3889 | 0.956 | 0.864 | 1.058 |
| *Definition of abbreviation*: SE=standard error; CI=confident interval.  Stepwise variable selection procedure was adopted to eliminate one-by-one those variables (other than the main exposure variable) with p-value over 0.25. | | | | | | |
