# Supplementary material for: Association between afterhours admission to the intensive care unit, strained capacity, and mortality: a retrospective cohort study
Source: Crit Care. 2018 Apr 17;22:97. doi: 10.1186/s13054-018-2027-8 (PMC5905119; doi:10.1186/s13054-018-2027-8)
Supplement: Supplementary file 7 — Multivariate, mixed effects logistic regression on hospital mortality. (DOCX 21 kb) [file 13054_2018_2027_MOESM7_ESM.docx]

**Additional File 7.** Multivariate, mixed effects logistic regression of hospital mortality.

| **Effect** | **Estimate** | **SE** | **p-value** | **OR 95% CI** | | |
| --- | --- | --- | --- | --- | --- | --- |
|  |  |  |  | **OR** | **LCL** | **UCL** |
| **Intercept** | -11.4206 | 0.3750 | <.0001 |  |  |  |
| **Age** |  |  |  |  |  |  |
| < 65 years | reference |  |  |  |  |  |
| 65-74 years | 0.2123 | 0.0561 | 0.0002 | 1.237 | 1.108 | 1.380 |
| 75-84 years | 0.4150 | 0.0605 | <.0001 | 1.514 | 1.345 | 1.705 |
| ≥ 85 years | 0.9052 | 0.0863 | <.0001 | 2.472 | 2.088 | 2.928 |
| **Hospital type** |  |  |  |  |  |  |
| Academic | reference |  |  |  |  |  |
| Community | 1.5560 | 0.4321 | 0.0120 | 4.740 | 2.032 | 11.055 |
| Tertiary | 0.4625 | 0.5130 | 0.4041 | 1.588 | 0.581 | 4.340 |
| **System** |  |  |  |  |  |  |
| Cardiovascular | reference |  |  |  |  |  |
| Gastrointestinal | -0.1103 | 0.0800 | 0.1681 | 0.896 | 0.766 | 1.048 |
| Genitourinary | -0.6909 | 0.1567 | <.0001 | 0.501 | 0.369 | 0.681 |
| Hematology | 0.2345 | 0.2584 | 0.3642 | 1.264 | 0.762 | 2.098 |
| Metabolic/Endocrine | -1.1208 | 0.2629 | <.0001 | 0.326 | 0.195 | 0.546 |
| Musculoskeletal/Skin | -0.1320 | 0.1327 | 0.3199 | 0.876 | 0.676 | 1.137 |
| Neurologic | -0.6470 | 0.1023 | <.0001 | 0.524 | 0.428 | 0.640 |
| Respiratory | -0.0653 | 0.0616 | 0.2887 | 0.937 | 0.830 | 1.057 |
| Transplant | -1.0071 | 0.3921 | 0.0102 | 0.365 | 0.169 | 0.788 |
| Trauma | -0.5586 | 0.1539 | 0.0003 | 0.572 | 0.423 | 0.773 |
| **Surgery** |  |  |  |  |  |  |
| Non-operative | reference |  |  |  |  |  |
| Elective | -0.8844 | 0.1546 | <.0001 | 0.413 | 0.305 | 0.559 |
| Emergent | -0.1378 | 0.0993 | 0.1652 | 0.871 | 0.717 | 1.058 |
| **Class** |  |  |  |  |  |  |
| Medical | reference |  |  |  |  |  |
| Neurological | 0.8701 | 0.1035 | <.0001 | 2.387 | 1.949 | 2.924 |
| Surgical | 0.0880 | 0.0970 | 0.3644 | 1.092 | 0.903 | 1.321 |
| Trauma without head injury | -0.1156 | 0.2026 | 0.5684 | 0.891 | 0.599 | 1.325 |
| Trauma with head injury | 0.7019 | 0.1702 | <.0001 | 2.018 | 1.445 | 2.816 |
| **Comorbidity** |  |  |  |  |  |  |
| Hepatic | 0.2887 | 0.0699 | <.0001 | 1.335 | 1.164 | 1.531 |
| Metastatic/ Leukemia/ Lymphoma | 0.2886 | 0.0737 | <.0001 | 1.335 | 1.155 | 1.542 |
| Cardiovascular | 0.1475 | 0.0532 | 0.0056 | 1.159 | 1.044 | 1.286 |
| Cirrhosis | 0.1869 | 0.0857 | 0.0291 | 1.206 | 1.019 | 1.426 |
| Digestive | 0.0967 | 0.0647 | 0.1353 | 1.101 | 0.970 | 1.250 |
| **Charlson Index** | 0.1084 | 0.0185 | <.0001 | 1.114 | 1.075 | 1.156 |
| **Admission APACHE II score** | 0.0658 | 0.0027 | <.0001 | 1.068 | 1.062 | 1.074 |
| **Afterhours admission** | -0.0576 | 0.0457 | 0.2074 | 0.944 | 0.863 | 1.032 |
| *Definition of abbreviation*: SE=standard error; CI=confident interval.  Stepwise variable selection procedure was adopted to eliminate one-by-one those variables (other than the main exposure variable) with p-value over 0.25. | | | | | | |
