# Supplementary material for: Association between afterhours admission to the intensive care unit, strained capacity, and mortality: a retrospective cohort study
Source: Crit Care. 2018 Apr 17;22:97. doi: 10.1186/s13054-018-2027-8 (PMC5905119; doi:10.1186/s13054-018-2027-8)
Supplement: Supplementary file 8 — Direct effects of admission APACHE II score on ICU mortality, hospital mortality, and length of stay. (DOCX 18 kb) [file 13054_2018_2027_MOESM8_ESM.docx]

**Additional File 8.** Direct effects of admission APACHE II score on ICU mortality, hospital mortality, and length of stay

| **Outcome** | **Direct Effect** | |
| --- | --- | --- |
| **Mortality** | **OR (95% CI)** | **p-value** |
| Death in ICU | 1.091 (1.084, 1.098) | <.0001 |
| Death within 30 hours in ICU | 1.138 (1.124, 1.151) | <.0001 |
| Death within 3 days in ICU | 1.117 (1.107, 1.127) | <.0001 |
| Death within 7 days in ICU | 1.108 (1.099, 1.117) | <.0001 |
| Death in hospital | 1.068 (1.062, 1.074) | <.0001 |
| **Length of Stay** |  |  |
| LOS in ICU | 1.020 (1.016, 1.024) | <.0001 |
| LOS in hospital | 1.007 (1.001, 1.013) | 0.0213 |
| *Definition of abbreviation*: ICU=intensive care unit; OR=odds ratio; CI=confident interval.  Stepwise variable selection procedure was adopted to eliminate one-by one those variables (other than the main exposure variable) with p-value over 0.25. | | |
