# Supplementary material for: Association between afterhours admission to the intensive care unit, strained capacity, and mortality: a retrospective cohort study
Source: Crit Care. 2018 Apr 17;22:97. doi: 10.1186/s13054-018-2027-8 (PMC5905119; doi:10.1186/s13054-018-2027-8)
Supplement: Supplementary file 9 — Multivariate, mixed-effects Poisson regression model for hospital length of stay. (DOCX 20 kb) [file 13054_2018_2027_MOESM9_ESM.docx]

**Additional File 9.** Multivariate, mixed effects Poisson regression model for hospital length of stay.

| **Effect** | **Estimate** | **SE** | **p-value** | **OR 95% CI** | | |
| --- | --- | --- | --- | --- | --- | --- |
|  |  |  |  | **OR** | **LCL** | **UCL** |
| **Intercept** | 2.5158 | 0.1558 | <.0001 |  |  |  |
| **Hospital type** |  |  |  |  |  |  |
| Academic | reference |  |  |  |  |  |
| Community | -0.3215 | 0.0727 | <.0001 | 0.725 | 0.629 | 0.836 |
| Tertiary | -0.0207 | 0.0923 | 0.822 | 0.98 | 0.817 | 1.174 |
| **Surgery** |  |  |  |  |  |  |
| Non-operative | reference |  |  |  |  |  |
| Elective | -0.1908 | 0.0325 | <.0001 | 0.826 | 0.775 | 0.881 |
| Emergent | -0.0689 | 0.1071 | 0.520 | 0.933 | 0.757 | 1.152 |
| **Class** |  |  |  |  |  |  |
| Medical | reference |  |  |  |  |  |
| Neurological | 0.4111 | 0.0768 | <.0001 | 1.508 | 1.298 | 1.753 |
| Surgical | 0.2399 | 0.0562 | <.0001 | 1.271 | 1.138 | 1.419 |
| Trauma without head injury | 0.4855 | 0.0141 | <.0001 | 1.625 | 1.581 | 1.67 |
| Trauma with head injury | 0.7276 | 0.0309 | <.0001 | 2.07 | 1.949 | 2.199 |
| **Comorbidity** |  |  |  |  |  |  |
| Chronic Dialysis | 0.1490 | 0.0547 | 0.0070 | 1.161 | 1.043 | 1.292 |
| Hepatic | -0.1916 | 0.0816 | 0.0190 | 0.826 | 0.704 | 0.969 |
| Cardiovascular | 0.0434 | 0.0306 | 0.1570 | 1.044 | 0.984 | 1.109 |
| Immune Suppression | 0.2327 | 0.0484 | <.0001 | 1.262 | 1.148 | 1.387 |
| Diabetes | -0.0759 | 0.0280 | 0.0068 | 0.927 | 0.877 | 0.979 |
| Digestive | 0.2036 | 0.0637 | 0.0014 | 1.226 | 1.082 | 1.389 |
| **Charlson Index** | 0.1669 | 0.0274 | <.0001 | 1.182 | 1.12 | 1.247 |
| **Occupancy** | 0.0035 | 0.0015 | 0.0172 | 1.004 | 1.001 | 1.007 |
| **Admission APACHE II score** | 0.0070 | 0.0026 | 0.0062 | 1.007 | 1.002 | 1.012 |
| **Afterhours admission** | -0.0423 | 0.0340 | 0.2140 | 0.959 | 0.897 | 1.025 |
| *Definition of abbreviation*: SE=standard error; CI=confident interval; LOS=length of stay.  Stepwise variable selection procedure was adopted to eliminate one-by-one those variables (other than the main exposure variable) with p-value over 0.25. | | | | | | |
